# Effect of a Ketogenic Medium Chain Triglyceride-Enriched Diet on the Fecal Microbiota in Canine Idiopathic Epilepsy: A Pilot Study

**DOI:** 10.3390/vetsci10040245

**Published:** 2023-03-24

**Authors:** Sylvia García-Belenguer, Laura Grasa, Jorge Palacio, Jon Moral, Belén Rosado

**Affiliations:** 1 Departamento de Patología Animal, Facultad de Veterinaria, Universidad de Zaragoza, Miguel Servet, 177, 50013 Zaragoza, Spain; sgarcia@unizar.es (S.G.-B.);; 2 Departamento de Farmacología, Fisiología y Medicina Legal y Forense, Facultad de Veterinaria, Universidad de Zaragoza, Miguel Servet, 177, 50013 Zaragoza, Spain; 3 Instituto de Investigación Sanitaria de Aragón (IIS), 50009 Zaragoza, Spain; 4 Instituto Agroalimentario de Aragón—IA2, Universidad de Zaragoza—CITA, 50009 Zaragoza, Spain

**Keywords:** dog, epilepsy, ketogenic diet, medium chain triglycerides, microbiota

## Abstract

**Simple Summary:**

Epilepsy is the most common neurological disorder in dogs. It is increasingly recognized that ketogenic diets enriched with medium chain triglycerides (MCT) have a positive impact on dogs with idiopathic epilepsy by reducing the frequency and severity of seizure activity. Significant evidence points towards a relationship between gut microbiota and epilepsy, and that the MCT diet may alter this microbiota. The current study examined the effects of a MCT diet administered for one month on the fecal microbiota in dogs with IE and non-epileptic beagles. The diet reduced Actinobacteria in all dogs while reducing seizure frequency in epileptic ones. Different baseline microbiota patterns were found in dogs with drug-sensitive epilepsy (DSE) and dogs with drug-refractory epilepsy (DRE). The baseline microbiota pattern of dogs with DSE was similar to that of non-epileptic dogs. In them, the MCT diet decreased the relative abundance of bacteria from the Firmicutes phylum and increased that of the Bacteroidetes and Fusobacteria phylum. The opposite effect was found in dogs with DRE. These results suggest that the MCT diet could help reduce gut microbiota differences between dogs with DRE or DSE.

**Abstract:**

Ketogenic diets have been successfully used in people and dogs with idiopathic epilepsy. This study examined the effect of a ketogenic medium chain triglycerides (MCT)- enriched diet administered for one month on the fecal microbiota of epileptic (*n* = 11) (six with drug-sensitive epilepsy, DSE; five with drug-refractory epilepsy, DRE) and non-epileptic beagle dogs (*n* = 12). A significant reduction after diet in the relative abundance of bacteria from the Actinobacteria phylum was observed in all dogs. Epileptic dogs showed a higher relative abundance of *Lactobacillus* compared with non-epileptic dogs at baseline but these differences disappeared after diet. Epileptic dogs also showed a significantly higher abundance of *Negativicutes* and *Selenomonadales* after dietary intervention. Baseline microbiota patterns were similar in non-epileptic beagles and dogs with DSE but significantly different from dogs with DRE. In non-epileptic and DSE groups, the MCT diet decreased the relative abundance of Firmicutes and increased that of Bacteroidetes and Fusobacteria, but the opposite effect was observed in dogs with DRE. These results suggest that the MCT diet effect would depend on individual baseline microbiota patterns and that ketogenic diets could help reduce gut microbiota differences between dogs with DRE and DSE.

## 1. Introduction

Epilepsy is the most common neurological disorder in humans and dogs with an estimated prevalence of 0.52–0.88% in humans [1,2] and 0.62–0.82% in dogs [3,4]. Around 50% of human and canine epileptic patients are diagnosed with idiopathic epilepsy (IE) [1,5,6], 30–40% of which are resistant to treatment with antiepileptic drugs (AED) [7]. Therefore, research on alternative or complementary therapeutic options for refractory epilepsy is of great clinical interest in both species.

People and animals with epilepsy show perturbed metabolic processing of glucose in epileptogenic brain areas, and the ketogenic diet (KD) provides ketone bodies that can be used as auxiliary brain fuel in addition to glucose [8]. Adding medium chain triglycerides (MCT) to the diet provides ketone bodies and octanoic and decanoic acids, which can be beneficial for reducing seizure frequency [8]. Importantly, besides brain energy support, KDs also have anti-inflammatory properties [9,10] and prevent neuronal hyperexcitability (reviewed by Berk et al. [11]). 

Dietary supplementation with MCT oil in commercially produced diets or added to food is increasingly recognized as having a positive impact on epileptic dogs by reducing the frequency and severity of seizure activity [12,13,14,15,16]. MCT-enriched diets have also been demonstrated to improve cognition in aged [17] and epileptic dogs [18], as well as improving anxiety-related signs [13].

Although evidence of a connection between gut microbiota and epilepsy has been recorded in human and veterinary medicine, further investigations are needed [19,20]. The therapeutic effect of KD in refractory epilepsy has been widely described although its efficacy and side effects vary depending on the specific characteristics of the KD [21]. Gong et al. [22], for instance, observed a significantly higher fecal microbial alpha diversity and higher abundance in Actinobacteria at the phylum level and *Enterococcus*, *Anaerostipes*, *Bifidobacterium*, *Bacteroides*, and *Blautia* at the genus level in children with drug-refractory epilepsy (DRE) compared to matched healthy controls. However, the abundance of these genera was reversed after 6 months of KD treatment, thus concluding that intestinal dysbiosis could be involved in the pathogenesis of DRE in children. Furthermore, Olson et al. [23] demonstrated that the KD altered the gut microbiota across two seizure mouse models for DRE and that these changes in microbiota, which were accompanied by elevated hippocampal gamma-aminobutyric acid (GABA)/glutamate levels, were necessary and sufficient for conferring seizure protection. In dogs with IE, Pilla et al. [24] observed that the ketogenic MCT diet resulted in a significant reduction in seizures frequency together with a significant increase in their fecal alpha diversity associated with a higher abundance of *Bacteroidaceae* compared to a baseline diet (home owner-managed diet).

Significant evidence, therefore, points towards a relationship between gut microbiota and epilepsy, and that the KD can alter this microbiota and reduce seizure frequency. However, different taxonomic shifts have been observed, highlighting that the KD-induced microbiota likely depends on host genetics and baseline microbiota profiles. There is also variability in the results in terms of which specific taxa and relative abundance of bacteria protect against seizures.

This study aimed to investigate the effect of a ketogenic MCT-enriched diet on the microbiota in canine IE. Changes in the phylogenetic composition and structure of the fecal microbiota from epileptic dogs were evaluated both before and after administering the diet for 30 days. Differences in the microbiota profiles between good responders to a single AED (DSE: drug-sensitive epileptic group) and those showing refractory epilepsy (DRE: drug-refractory epileptic group) were also analyzed. In parallel, the effect of the same KD on the fecal microbiota of a homogeneous population of non-epileptic dogs was studied and compared results with those of epileptic patients.

## 2. Materials and Methods

### 2.1. Animals and Procedures

This pilot study was a non-blinded, no placebo, prospective clinical trial conducted in two groups, sick (epileptic) and healthy (non-epileptic) dogs. The epileptic dogs were recruited from the neurology service of our veterinary teaching hospital (Hospital Veterinario Universidad de Zaragoza). To be included in the study, dogs had to have been diagnosed with IE according to the International Veterinary Epilepsy Task Force (IVETF) Level I confidence level criteria [25] at least 6 months before the start of the study and be on stable pharmacological treatment. In addition, the dogs had to be free of any other pathology (normal physical and neurological examination and normal laboratory analysis), they had to be correctly vaccinated and dewormed, and their feces had to have a normal appearance. 

The non-epileptic dogs were a group of beagles owned by the University of Zaragoza (Veterinary Faculty) for research purposes. To be included in the study they had to be healthy (normal physical and neurological examination and normal laboratory analysis), they had also to be correctly vaccinated and dewormed, and their feces had to have a normal appearance. 

EI was defined according to IVETF [26] and characterized by an enduring predisposition to generate generalized epileptic seizures, with seizures onset between 6 months and 6 years old and normal interictal neurological exam. Epileptic dogs were classified as having DSE (Drug-Sensitive Epilepsy), when they were being treated with one single AED (phenobarbital or imepitoin) with a good clinical response for at least the previous 3 months, or with DRE (Drug-Refractory Epilepsy), when they were under multi-drug treatment. DRE is defined by the International League against Epilepsy as a failure to achieve sustained seizure freedom after at least two appropriate AED trials [7]. To include dogs with IE in this group, the maximum dose of phenobarbital (30–35 mg/L serum) or imepitoin (30 mg/kg/12 h) had been reached and a combination with KBr (10–20 mg/kg/12 h) or levetiracetam (20–30 mg/kg/8 h) had been required, following consensus criteria from the International Veterinary Epilepsy Task Force (IVETF) [27]. In addition, dogs in the DRE group had to have a normal encephalic MRI in their diagnostic history.

During the study period, there were no changes in handling other than diet. Changes in treatment or any other problem during the development of the study constituted exclusion criteria.

#### Ethics Statement

Before enrollment, owners were informed about the study and procedures. The selected diet for this study is commercially available for canine IE and cognitive dysfunction and its administration was always indicated by clinical criteria as part of the treatment. Owners were allowed the opportunity to ask any questions and to confirm or decline participation. All procedures were carried out under Project License PI27/18 approved on 17 July 2018 by the Ethics Committee for Animal Experiments from the University of Zaragoza. The care and use of control dogs were performed following the Spanish Policy for Animal Protection RD53/2013, which meets the European Union Directive 2010/63 on the protection of animals used for experimental and other scientific purposes.

### 2.2. Diet

For the study, all dogs were fed a commercially produced KD enriched in MCT (Nestlé Purina^®^PetCare, ProPlan^®^ Veterinary Diets, Barcelona, Spain). This diet was progressively introduced throughout a week. After this week of adaptation, all the dogs were fed exclusively with the new diet for one month. This new diet consisted of a high MCT oil (6.5%)—diet. Analytical constituents included 30% crude protein, 15% crude fat (MCT included), 7.5% crude ash, 1.5% crude fiber, 0.4% EPA + DHA, and 210 mg/kg vitamin B group.

### 2.3. Fecal Collection and Microbiota Analysis

Fecal samples (1–3 g) were collected from both groups before (D0) and after 1 month on the MCT diet (D30) directly from the rectal ampoule with sterile gloves and immediately frozen at −80 °C to fix bacterial growth and preserve DNA content.

Bacterial DNA extraction from fecal samples and sequencing of bacterial 16S rRNA gene procedures were the same as we previously published [28]. Bacterial DNA was extracted from fecal samples using the NZY Soil gDNA Isolation kit (NZYTech, Lisboa, Portugal). The V4 region of the 16S rRNA gene was amplified using specific primers (515F-806R) [29] with a barcode. All PCR reactions were carried out with Phusion High-Fidelity PCR Master Mix (New England Biolabs, Ipswich, MA, USA). Sequencing libraries were generated using NEBNext Ultra DNA Library Pre^®^ Kit for Illumina^®^ (New England Biolabs, Ipswich, MA, USA). The library was then sequenced on an Illumina MiSeq platform and 250 bp paired-end reads were generated.

### 2.4. Bioinformatics and Statistical Methods

Bioinformatic analyses were the same as previously published [25]. Paired-end reads were merged and filtered using QIIME v.1.7. Sequences with ≥97% similarity were assigned to the same operational taxonomic units (OTUs). The SILVA Database was used for species annotation at each taxonomic rank. Analysis of alpha and beta diversity were calculated with QIIME and displayed with R software (v.2.15.3). The Observed Species, Chao1, and the Shannon biodiversity indices were used to estimate alpha diversity (intra-individual diversity). Non-metric multidimensional scaling (NMDS) plots and analysis of similarity (Anosim) were used to estimate beta diversity.

A one-tailed Wilcoxon matched-paired signed ranks test was used to compare alpha-diversity, the relative abundance of bacteria at different taxonomic levels, and clinical outcomes between two moments in time (D0 and D30) both in the epileptic and non-epileptic groups, as well as in the DSE and DRE-groups. A one-tailed Mann-Whitney U test was used to compare these variables between epileptic and non-epileptic dogs at D0 to rule out any potential biases, and at D30, when they were all with the same diet. In addition, a principal component analysis (PCA) was performed to reduce the larger set of bacteria (variables) into a smaller set which accounted for most of the variance. An unrotated factor solution was used and variables with eigenvalues >0.7 were extracted. The proportion of total variance accounting for the three first components was 55.7%. Finally, a multifactorial multivariate analysis of variance with repeated measures was carried out to assess the disease (IE) and diet (MCT-diet) effects and their interaction with the selected bacteria at different taxonomic levels. Statistical analyses were carried out with IBM SPSS 19.0 for Windows, and the type I error (*p*) was set at 0.05.

## 3. Results

### 3.1. Clinical Outcomes

Table 1 shows the demographic and clinical data in epileptic and non-epileptic dogs. For the epileptic group, 14 dogs were recruited but three dogs withdrew from the study due to poor acceptance of the diet and only 11 dogs concluded the study (six females and five males). This group was made up of dogs of different breeds (three Border collies, one Maltese, one San Bernardo, one small-sized and five medium-sized mongrel dogs), aged between 2–9 years old and weighing between 3.4–68.0 kg. Even it was a very heterogeneous group in terms of body weight, all the animals showed good body condition (mean body score: 5/9), and suffered no changes in their weight and body condition at the end of the trial. They all had an owner and lived in an urban environment in the same city.

Six of the epileptic dogs were classified with DSE, with three of them being treated with phenobarbital (dose range 2.5–4 mg/kg/12 h) and the remaining with imepitoin (dose range 10–20 mg/kg/12 h). The other five dogs were classified as having DRE, and were under different combinations of phenobarbital, imepitoin, KBr, and levetiracetam.

A significantly lower seizure frequency was observed during the month of the study with the MCT-diet compared to the frequency in the previous month (2.2 ± 1.6 vs. 1.3 ± 1.3 seizures/month, *p* = 0.015). When the response to AED was considered, this signification remained only as a trend in both the DSE (*p* = 0.083) and the DRE (*p* = 0.059) groups. Of the six dogs with DSE, four achieved seizure freedom at the end of the study, and two animals remained at the same seizure frequency as at baseline. Of the five dogs with DRE, four showed a reduction in monthly seizure frequency during the period of study (50% reduction in three of them), one dog exhibited <50% reduction, and one dog maintained initial seizure frequency.

The non-epileptic group consisted of 12 healthy beagles (seven females and five males), aged between 2–9 years old, and weighing between 11.2–17.0 kg. All of them started from a fairly homogeneous weight and they showed a significant (*p* = 0.003) weight reduction after one month on the new diet, along with a significant body condition reduction (5.2/9 vs. 4.5/9, *p* = 0.005). Half of these dogs usually (30–50% of the study days) did not finish the food ration corresponding to their weight.

At the time of the study, epileptic dogs were fed by their owners a commercial maintenance food with a different composition (range of composition: 22–30% crude protein, 7–18% crude fat, 5.3–10.5% crude ash, 1.3–10% crude fiber). Similarly, non-epileptic dogs were fed by their caregivers a commercial maintenance diet (25% crude protein, 14% crude fat, 6.1% crude ash, 1.3% crude fiber), given in amounts to cover maintenance energy requirements.

### 3.2. Differences in Fecal Microbiota between Epileptic and Non-Epileptic Dogs before and after MCT Diet

**Figure 1:**
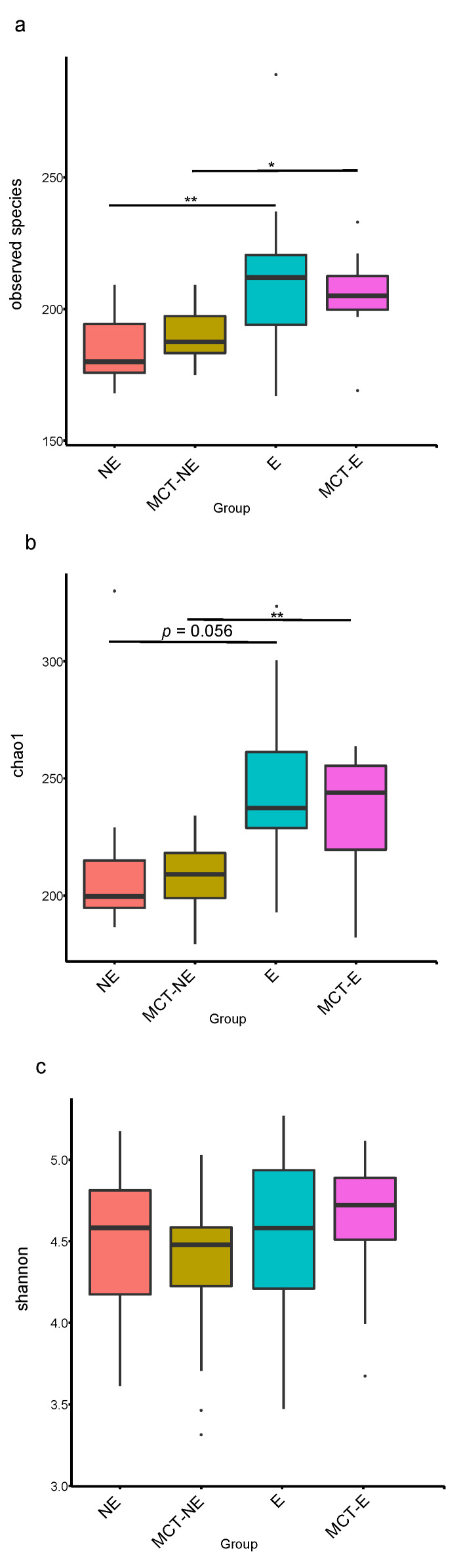
Fecal alpha diversity indices: (**a**) Observed Species; (**b**) Chao1; (**c**) Shannon, measured in epileptic (E) and non-epileptic (NE) dogs before (D0) and after the MCT diet (D30). Asterisks indicate significance between groups (* *p* < 0.05; ** *p* < 0.01).

Alpha diversity indices were higher in epileptic than in non-epileptic dogs (Figure 1). The number of Observed Species was significantly higher in epileptic than in non-epileptic dogs, both before (*p* = 0.009) and after (*p* = 0.013) the MCT diet. Similarly, the Chao1 non-parametric estimator of species richness was significantly higher in epileptic than in non-epileptic dogs, both before (*p* = 0.011) and after (*p* = 0.006) the MCT diet. There were no significant differences between groups in the Shannon index.

**Figure 2:**
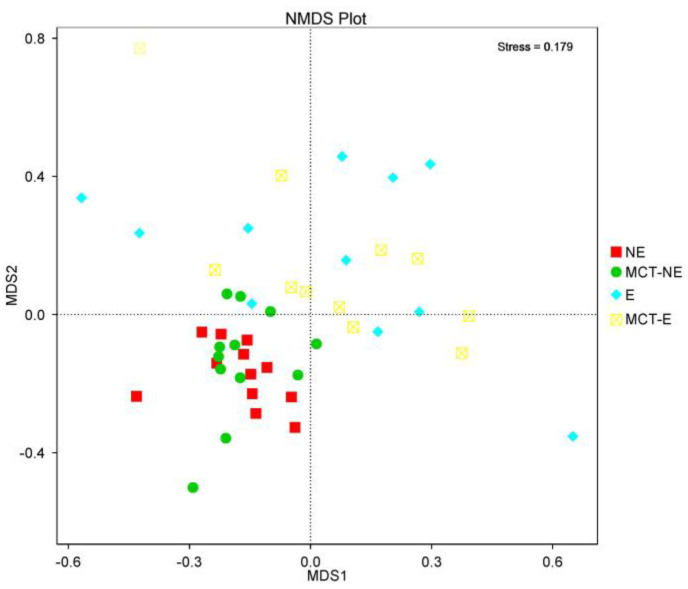
Non-metric multidimensional scaling (NMDS) plots showed that epileptic (E) and non-epileptic (NE) dogs were two distinct clusters before and after the MCT diet.

**Figure 3:**
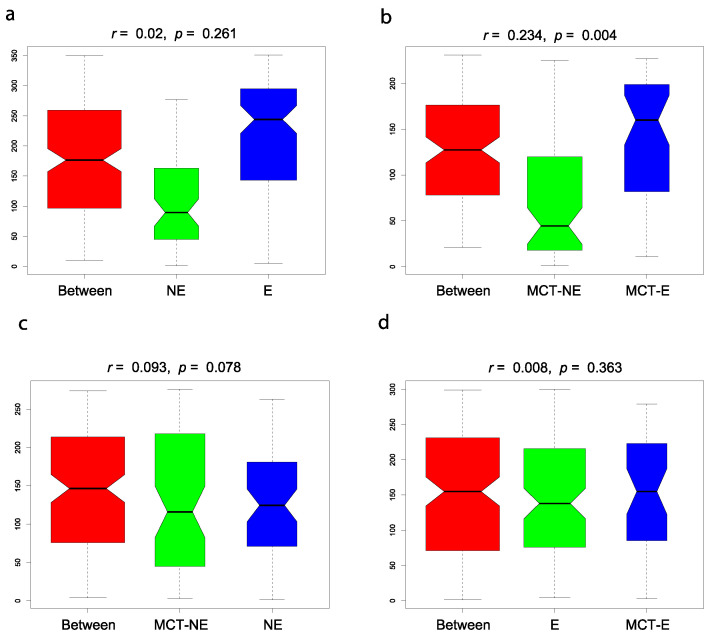
Analysis of similarity (Anosim) between epileptic (E) and non-epileptic (NE) dogs in baseline conditions ((**a**): E vs. NE) (Anosim *r* = 0.002; *p* = 0.261), between E and NE dogs after the MCT diet ((**b**): MCT-E vs. MCT-NE) (Anosim *r* = 0.234; *p* = 0.004), in NE dogs before and after the MCT diet ((**c**): NE vs. MCT-NE) (Anosim *r* = 0.093; *p* = 0.078) and E dogs before and after the MCT diet ((**d**): E vs. MCT-E) (Anosim *r* = 0.008; *p* = 0.363).

Regarding beta diversity, non-metric multidimensional scaling (NMDS) plots showed a different cluster for epileptic and non-epileptic dogs (Figure 2). Analysis of similarity (Anosim) revealed significant differences between epileptic and non-epileptic dogs but only after the MCT diet (Anosim *r* = 0.234; *p* = 0.004) (Figure 3).

Concerning the relative abundance of fecal bacteria in basal conditions (D0), dogs with IE showed a higher abundance of *Bacilli* (*p* = 0.006) class, *Lactobacillales* (*p* = 0.041) order, and *Lactobacillus* (*p* = 0.004) genus compared to non-epileptic dogs. After 1 month of dietary intervention (D30), epileptic dogs showed a significantly higher abundance of *Negativicutes* (*p* = 0.000) and *Bacilli* (*p* = 0.019) at the class level, *Selenomonadales* (*p* = 0.000) at the order level, *Veillonellaceae* (*p* = 0.000) at the family level and *Megamonas* (*p* = 0.000) at the genus level, and lower abundance of *Peptostreptococaceae* (*p* = 0.044) at the family level and *Peptoclostridum* (*p* = 0.009) at the genus level than non-epileptic dogs.

### 3.3. Fecal Microbiota Results in Epileptic Dogs before and after MCT Diet

The introduction of the MTC diet in this group of dogs did not modify the alpha (Figure 1) or beta (Figure 2 and Figure 3) diversity compared to their baseline condition (D0 vs. D30), but a significant reduction was observed in the relative abundance of bacteria from Actinobacteria (*p* = 0.004) phylum, *Coriobacteria* (*p* = 0.008) class, and *Coriobacteriales* (*p* = 0.008) order.

### 3.4. Fecal Microbiota Results in Non-Epileptic Dogs before and after MCT Diet

The introduction of the MTC diet in this group of dogs did not modify the alpha (Figure 1) or beta (Figure 2 and Figure 3) diversity compared to their baseline condition (D0 vs. D30), but a significant reduction was observed in the relative abundance of bacteria from Actinobacteria (*p* = 0.006) phylum, *Negativicutes* (*p* = 0.002) and *Coriobacteria* (*p* = 0.004) classes and *Ruminococcus gnavus_group* (*p* = 0.002) and *Megamonas* (*p* = 0.002) genera. 

### 3.5. Differences in Fecal Microbiota between Drug-Refractory and Drug-Sensitive Epileptic Dogs

No significant differences were found in alpha diversity or beta diversity between the DRE and DSE groups, but there were some differences in the relative abundance of several bacteria at baseline (D0). Thus, dogs with DRE, compared to dogs with DSE, showed a higher relative abundance of *Bacteroidia* (*p* = 0.012) and *Fusobacteria* (*p* = 0.042) classes, as well as a lower relative abundance of Firmicutes (*p* = 0.019) phylum and *Clostridia* (*p* = 0.019) class, highlighting the reduction in the *Blautia* (*p* = 0.002) genus. After the MCT diet, the microbiota of the DRE and DSE groups tended to equalize and no significant differences were found in the relative abundance of bacteria.

**Figure 4:**
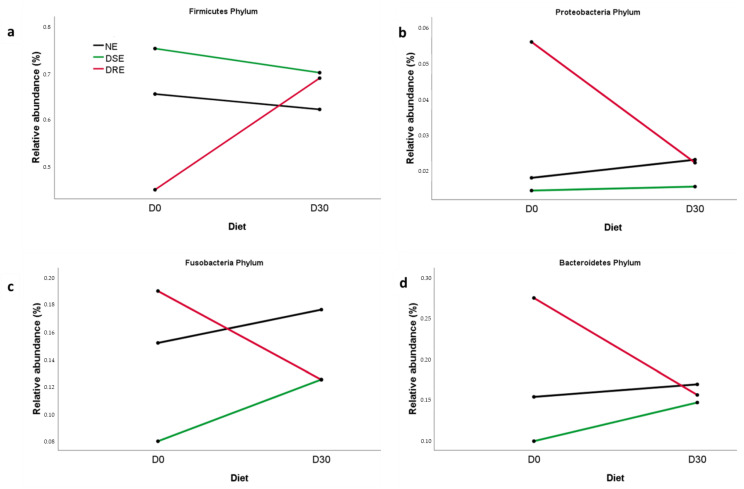
Means of (**a**) Firmicutes, (**b**) Proteobacteria, (**c**) Fusobacteria, and (**d**) Bacteroidetes at phylum level for non-epileptic (NE), drug-sensitive epilepsy (DSE) and drug-refractory epilepsy (DRE) groups, before (D0) and after one month on the MCT diet (D30).

The selected bacteria after PCA were Firmicutes, Proteobacteria, Bacteroidetes, and Fusobacteria at the phylum level, *Clostridia*, *Negativicutes*, *Gammaproteobacteria*, *Bacteroidiia*, *Fusobacteriia* and *Betaproteobacteria* at class level, *Clostridiales*, *Selenomonadales*, *Enterobacteriales*, *Bacteroidales*, *Fusobacteriales*, and *Bukholderiales* at the order level, *Lachnospiraceae*, *Enterobacteriaceae*, *Villonallaceae*, *Fusobacteriaceae* and *Bacteriaceae* at the family level and *Blautia*, *Megamonas*, *Bacteroides*, and *Fusobacterium* at the genus level. When the multivariate analysis was applied, there were found significant differences between non-epileptic, DRE, and DSE groups at the phylum level with a significant diet effect (*p* = 0.048). Multifactorial multivariate analysis of variance with repeated measures did not find interactions between disease and diet, but at the univariate level, the phylum Firmicutes, Fusobacteria, Proteobacteria, and Bacteroidetes showed a significant interaction (*p* < 0.05), mainly due to the different microbiota patterns in the DRE group (Figure 4).

## 4. Discussion

Clinically, there were no adverse effects for the non-epileptic dogs with the administration of a ketogenic MCT-enriched diet for one month. However, they showed a significant loss of weight and body condition, probably related to poor acceptance of diet in some animals, but perhaps also due to the KD effect in metabolic control. In this sense, KD has been shown to be effective in reducing weight in human patients with overweight or obesity, especially in those with preexisting diabetes [30]. In any case, extending the use of the MCT diet in these healthy dogs would not have been recommended.

On the contrary, epileptic dogs stayed at their starting weight and showed a significant reduction in seizure frequency. This reduction cannot be directly associated with the introduction of the MCT-diet, since one month is a short time to assess the evolution of the seizures, taking into account the episodic nature of epilepsy with normal ups and downs. However, previous studies support the effect of KD in reducing seizure frequency both in humans [8,22] and dogs [8,12,15,16,24].

In humans, KD is beneficial in certain types of epilepsy as long as patients can tolerate and maintain these dietary regimens [8]. The lack of tolerance to this type of diet can also be a problem in the canine species. However, a recent study comparing the palatability and tolerance of an MCT oil or a tasteless control oil as a dietary supplement found no differences in the average food intake or intake ratio between food with and without oil supplementation or between the two oil groups, although the mean food intake time was longer in the group that received the MCT-oil [31]. In any case, the better acceptance of the diet in the group of epileptic dogs could also be related to an increase in appetite associated with AED treatment, since polyphagia is a frequent adverse effect of most of these drugs [27].

In our study, a significant reduction in the relative abundance of bacteria from the Actinobacteria phylum was observed in both epileptic and non-epileptic dogs after 30 days on the MCT diet. A higher abundance of Actinobacteria has been described in children with DRE but these bacteria were significantly reduced after six months on a KD [22]. Recently, another study in children with DRE treated with a KD for three months found a reduction of the TNF (Tumor Necrosis Factor) and specific Bifidobacteria that belong to the Actinobacteria phylum. The authors of the study concluded that epileptic children with a higher abundance of members of the *B. longum* cluster and higher levels of TNF may be more likely to benefit from KD treatment and suggested that both factors might be useful biomarkers to identify potential responders to KD before treatment initiation [10]. Taken together, our results and those from human studies suggest that Actinobacteria reduction could be a characteristic beneficial effect of KD in epileptic patients. On the other hand, Pilla et al. [24] identified an unnamed *Bacteroidaceae* species within genus 5-7N15 as a potential biomarker associated with the consumption of the MCT diet in epileptic dogs.

We found no differences in alpha or beta diversity attributable to diet when comparing biodiversity before and after one month with the ketogenic MCT-enriched diet in both epileptic and non-epileptic dogs. However, Pilla et al. [24] found that consuming a ketogenic MCT diet for three months significantly increased alpha diversity in epileptic dogs. On the other hand, we found differences in microbiota biodiversity that could be attributable to disease (IE). However, the results that are discussed below regarding the comparison between non-epileptics and epileptics dogs should be interpreted with caution as the study groups differed in several aspects. Thus, non-epileptic dogs were a homogenous group of healthy beagles that lived under the same environmental and handling conditions (university facilities with daily walks and human contact) and were fed the same diet. In contrast, the epileptic population consisted of owned dogs of different breeds and fed different foods at baseline, although they shared certain common characteristics such as disease (IE), age (young adults), body condition (good), and living in an urban environment. Despite these similarities, the beta diversity plot (Figure 2) showed a large dispersion of animals, highlighting the high diversity of microbiota within epileptic dogs. Once the MCT diet was administered for one month, the dispersion in the composition of microbiota within this group was reduced, becoming closer to that of the healthy group, although we still observed that non-epileptic and epileptic dogs were two different populations.

In the present study, alpha diversity was higher in epileptic dogs compared to non-epileptic dogs both before and after the MCT diet. Similarly, Gong et al. [22] observed a significantly higher alpha diversity in children with DRE compared to matched healthy controls. Our finding could be associated with the diversity of environmental characteristics of owned dogs. Heterogeneity in the microbiota of epileptic dogs could in turn lead to different responses to the diet and different effects on the regulation of the gut-brain axis, which could explain individual differences in seizure control after diet. Other studies also show that the MCT-enriched diet has a positive effect in canine IE but with a variable response on seizure control: some dogs show ≥50% reduction but others <50%, other dogs remain unchanged and some even show increased seizure frequency [12,15].

Interestingly, epileptic dogs showed a higher relative abundance of *Lactobacillus* genus at baseline compared to healthy beagles. Alterations in Lactobacilli populations in the gut have been linked to the development and progression of several neurological conditions and it has been recognized that they can produce GABA. Moreover, increased concentrations of this neurotransmitter in the gastrointestinal tract have been shown to correlate with increased levels in the central nervous system [32]. On the opposite, Muñana et al. [33] found no differences in the relative or absolute abundance of *Lactobacillus* in drug-naïve epileptic dogs when compared to healthy dogs. In our study, after one month on the MCT diet, the differences between epileptic and non-epileptic dogs in Lactobacilli populations disappeared, although the differences at the *Bacilli* class level remained. A recent study in rats with kainic acid-induced status epilepticus observed that treatment with probiotics containing Lactobacilli and Bifidobacteria ameliorated spontaneous seizures and cognitive deficits [34]. Considering this, the role of Latobacillli and Bifidobacteria in seizure control should be studied more in-depth.

Furthermore, after one month on the MCT diet, epileptic dogs showed a significantly higher abundance of *Negativicutes* class (belonging to the Firmicutes phylum) compared to non-epileptic dogs. In particular, an increase within this class of *Selenomonadales* order, *Veillonellaceae* family, and *Megamonas* genus was observed in dogs with IE. Recently, a low abundance of *Negativicutes* class and *Selenomonadales* order has been linked to Parkinson’s disease in rats, with an increase of these taxa being observed when rats were treated with Tianqi Pingchan Granule, a clinically effective formula of traditional Chinese medicine that attenuates the production of peripheral inflammatory cytokines and inhibits the activation of microglia and astrocytes in substantia nigra [35].

When considering the response to AED, dogs with DRE showed a lower relative abundance of bacteria included in the Firmicutes phylum and *Clostridia* class, as well as in the *Blautia* genus, compared with dogs with DSE before the introduction of diet. Despite the different disease-related microbiota patterns, these differences could be related to the higher phenobarbital doses usually used in DRE compared with DSE. In this line, Watanangura et al. [36] found a decrease in the order of *Clostridiales* after three months of phenobarbital treatment in epileptic dogs.

The MCT diet’s effect differed depending on the epileptic subgroup and their basal microbiota composition. Thus, dogs with DSE showed very similar basal characteristics to the group of healthy beagles and in both cases, the diet decreased the relative abundance of Firmicutes and increased that of Bacteroidetes and Fusobacteria. However, the diet had the opposite effect in dogs with DRE, significantly increasing Firmicutes and decreasing Bacteroidetes, Fusobacteria, and even Proteobacteria. These differences in microbiota profiles between dogs with DRE or DSE could help explain the lack of response of dogs with DRE to AED. Moreover, these results suggest that the MCT diet could help to reduce microbiota differences between dogs with DRE and DSE.

Some limitations of this study should be highlighted. First, the small number of subjects, especially when comparing the results of dogs diagnosed with DRE or DSE. Second, the fact of not having placebo group regarding the diet, since it was not possible to have an equivalent diet without MCT. Thus, this did not allow us to determine whether the observed changes in the gut microbiota were due to the MCTs or to the KD diet as a whole. In any case, the changes observed in the fecal microbiota after one month on the MCT-diet were consistent, especially in the group of healthy dogs, which started from a homogeneous diet and could contribute to the understanding of the effects of MCT-enriched diets. Finally, as already stated, the comparison between the groups of epileptic and non-epileptic dogs must be interpreted with caution, given the different characteristics of both groups. Thus, the homogeneity of the beagles group would make it possible to study the effect of the diet without the intervention of other factors under controlled environmental conditions, while the group of epileptic dogs could represent the real variability at the clinical level. Despite these limitations, this pilot study provides relevant information that can help guide further studies that are undoubtedly necessary to continue deepening our knowledge of the role of gut microbiota in the development of IE.

## 5. Conclusions

Similar to studies in epileptic children, a significant reduction in the relative abundance of bacteria from Actinobacteria phylum was observed in both the epileptic and non-epileptic groups after consumption of the MCT diet for 30 days, which could contribute to reducing seizures in the latter. Moreover, epileptic dogs showed a higher relative abundance of *Lactobacillus* at baseline compared with non-epileptic dogs, but this difference disappeared after the dietary intervention for one month. Epileptic dogs also showed a significantly higher abundance of *Negativicutes* class and *Selenomonadales* order compared to non-epileptic dogs after diet, two taxa recently involved in Parkinson´s disease. Considering these findings, these bacterial taxa (i.e Actinobacteria phylum, *Negativicutes* class, *Selenomonadales* order, and *Lactobacillus* genus) should be further investigated in canine IE as possible biomarkers associated with the response to the MCT diet.

When considering the response to AED, the MCT-diet effect varied depending on the baseline microbiota patterns. Thus, DSE dogs showed very similar basal microbiota characteristics to the group of non-epileptic dogs, and in both cases, the diet decreased the relative abundance of Firmicutes and increased that of Bacteroidetes and Fusobacteria. However, the diet had the opposite effect on DRE dogs. These preliminary results suggest that ketogenic MCT diets could help reduce differences in gut microbiota between dogs with DRE or DSE, but future studies with larger numbers of dogs will be required to confirm these findings.

Clinically, epileptic dogs showed good acceptance and tolerance of the MCT-enriched diet, which could be beneficial for seizure control.

## Figures and Tables

**Table 1:** Demographic and clinical data in the studied canine population.

Group	Number of Dogs	Males	Females	Age (Years)	Weight D0 (kg)	Weight D30 (kg)	Seizures D0 (per Month)	Seizures D30 (per Month)
		(%)		Mean ± SD	Mean ± SD	Mean ± SD	Mean ± SD	Mean ± SD
NE	(*n =* 12)	41.6	58.3	4.2 ± 2.9	14.9 ± 1.8 ^ab^	13.8 ± 2.2 ^ab^	Not applicable	Not applicable
E	(*n =* 11)	45.5	54.5	5.6 ± 1.9	22.2 ± 17.1 ^b^	22.2 ± 16.6 ^b^	2.2 ± 1.6 ^a^	1.3 ± 1.3 ^a^
DSE	(*n =* 6)	33.3	66.7	4.7 ± 1.8	24.0 ± 22.8	24.0 ± 22.2	0.8 ± 0.4	0.3 ± 0.5
DRE	(*n =* 5)	60.0	40.0	6.8 ± 1.8	20.0 ± 8.2	20.2 ± 8.3	3.8 ± 0.4	2.4 ± 0.9

NE, non-epileptic dogs; E, dogs with idiopathic epilepsy; DSE, dogs with drug-sensitive epilepsy; DRE, dogs with drug-refractory epilepsy; D0, day 0 before MCT-diet introduction; D30, day 30 after MCT diet introduction; SD, Standard deviation. ^a^ *p* < 0.05 for comparisons within groups (before and after the introduction of the MCT diet). ^b^ *p* < 0.05 for comparisons between different groups.

## Data Availability

The data of the sequences are available in NCBI Sequence Read Archive (SRA), BioProject ID PRJNA835309.
